# Associations of positive childhood experiences with disordered eating attitudes and behaviors among Korean college students

**DOI:** 10.3389/fpubh.2026.1817868

**Published:** 2026-07-15

**Authors:** Haemi Jun, Hyeeun Park, Seungha Shin, Yejin Heo, Cynthia Yursun Yoon

**Affiliations:** 1 Department of Food Science and Nutrition, Pusan National University, Busan, Republic of Korea; 2 Kimchi Research Institute, Pusan National University, Busan, Republic of Korea

**Keywords:** benevolent childhood experiences, childhood experiences, college students, cross-sectional study, disordered eating behaviors, positive childhood experiences

## Abstract

Positive childhood experiences (PCEs) are considered as early-life psychosocial resources which promote adaptive coping and reduce vulnerability to maladaptive health behaviors, including engagement in disordered eating attitudes and behaviors (DEABs). Although inverse associations between PCEs and DEABs have been documented among US college students, evidence from non-Western populations remains limited. This study examined associations between cumulative PCEs and multiple DEABs among 273 Korean college students (Mage = 22.7 ± 2.2; 71.4% female) in 2025. Independent variable PCEs included supportive relational and emotional experiences and were categorized into tertiles (T1 = lowest, T3 = highest). Dependent variable was DEABs, which includes overeating, binge eating, anxiety-associated symptoms of binge eating, unhealthy weight control behaviors, and body weight and shape concerns. Covariates included age, sex, academic classification, maximum parental educational attainment, and body mass index. Modified Poisson regression models estimated prevalence ratios (PRs) and 95% confidence intervals (CIs). Compared with students in T3, those in T2 and T1 had higher prevalence of binge eating (PR = 1.32 and 1.20 for T2 and T1, respectively) and body weight and shape concerns (PR = 1.55 and 1.53), although CIs included the null value. No clear dose-response pattern emerged. The selective associations suggest that the relationship between PCEs and DEABs may vary across specific behavioral domains and sociocultural contexts, and should be interpreted cautiously. These findings provide preliminary evidence consistent with life-course perspectives between supportive childhood environments to later eating-related behaviors, although the mechanisms underlying these associations remain unclear. Longitudinal research is warranted to clarify life-course pathways from positive early environments to eating-related health behaviors across sociocultural contexts.

## Introduction

Disordered eating attitudes and behaviors (DEABs), including overeating, binge eating, unhealthy weight control behaviors (e.g., fasting or purging), and excessive concerns about body weight and shape, are prevalent among college students (1) and are associated with adverse mental and physical health outcomes (2–4). As such, DEABs constitute an important public health concern during emerging adulthood. However, DEABs, encompassing a heterogeneous set of behaviors and attitudes rather than a single unified construct may suggest that different DEAB manifestations may arise through distinct psychological and behavioral pathways. For example, binge eating has frequently been conceptualized as a maladaptive emotion regulation strategy used to cope with psychological distress (5), whereas body weight and shape concerns are more closely related to self-concept development and internalization of sociocultural appearance ideals (6, 7). In contrast, unhealthy weight control behaviors may be more strongly influenced by dieting norms, peer environments, and cultural pressures surrounding body weight (8, 9). Life-course epidemiology posits that health-related behaviors in emerging adulthood are influenced by exposures and experiences occurring earlier in life that become biologically and behaviorally embedded across development (10, 11). Accumulation, sensitive periods, and pathways models suggest that childhood experiences can exert lasting effects on later health and behavioral outcomes through multiple mechanisms (10, 12–14). These frameworks provide a useful conceptual foundation for examining whether positive childhood experiences serve as early-life psychosocial resources that may protect against disordered eating attitudes and behaviors during emerging adulthood. However, research examining childhood experiences in relation to DEABs has been disproportionately focused on adverse childhood experiences (ACEs), including abuse, neglect, and household dysfunction) and documented ACEs associated with greater engagement in DEABs (10, 12, 14), which have been interpreted as reflecting disruptions in emotional regulation, self-concept, and stress coping processes (5). While this body of research has provided important insights into the long-term consequences of early childhood adversity, it emphasizes risk exposures and provides a limited understanding of how supportive childhood environments may contribute to resilience and healthier behavioral outcomes.

Positive childhood experiences (PCEs), another important dimension of childhood experiences, reflect the presence of stable, supportive, and nurturing relational environments during childhood, including having caring adults, predictable routines, and experience of safety and self-worth (15). Importantly, PCEs are conceptually distinct from the absence of adversity and may independently shape coping processes, emotional regulation, and interpersonal security (15). PCEs are commonly assessed using the Benevolent Childhood Experiences scale (15), which was developed to capture key domains of positive relational and environmental experiences during childhood, including supportive caregiving relationships, positive peer connections, and feelings of safety and belonging (15). These experiences represent psychosocial resources that may foster resilience by promoting adaptive coping strategies, emotional regulation capacities, and stable self-concept development. Emerging literature conceptualizes PCEs as protective developmental assets that may buffer the long-term health consequences of early-life stress and adversity (14, 16, 17). From a developmental perspective, early relational resources may shape how individuals respond to stress and regulate emotions later in life (18, 19) and such processes may be particularly relevant for maladaptive coping behaviors, including disordered eating (14). To that extent, supportive childhood environments that foster emotional security and adaptive coping may therefore be especially relevant for behaviors related to emotional regulation processes, such as binge eating, while associations with other behaviors may be weaker or more context-dependent. Nevertheless, empirical research examining PCEs and DEABs remains limited. Several recent studies have reported inverse, dose-dependent associations between cumulative PCEs and DEABs, suggesting that increasing exposure to supportive relational environments may incrementally reduce vulnerability to disordered eating behaviors. However, much of this evidence derives from cross-sectional designs, conducted primarily in the Western populations, particularly in the United States (14, 16, 17). Consequently, relatively little is known about whether similar associations operate in non-Western sociocultural contexts. To date, the few studies conducted in non-Western populations have documented that positive childhood experiences are related to outcomes such as family functioning and sleep quality (20), leaving eating-related behaviors largely unexplored. Nevertheless, cross-cultural examination in relation to eating-related behaviors is important because the expression and meaning of supportive childhood environments may vary across cultural settings. In Western contexts, parental warmth and support are often conveyed through explicit verbal affirmation and affective expression (21, 22), whereas in many East Asian contexts, including South Korea, support may be expressed more indirectly through instrumental provision, educational investment, and relational consistency (23, 24). Such culturally patterned expressions of care may influence how positive relational experiences are perceived, internalized, and integrated into emotional and behavioral regulation processes.

Cultural frameworks suggest that norms surrounding authority, family obligation, emotional expression, and interpersonal connectedness vary across societies and shape how individuals interpret and respond to relational experiences throughout development (25, 26). Theoretical perspectives from cross-cultural psychology further suggest that individuals in more interdependent cultural contexts tend to define the self through social relationships and obligations, whereas those in more independent contexts place greater emphasis on personal autonomy and individual emotional expression (27). Consequently, experiences that convey care, belonging, and relational security may be communicated and interpreted differently across cultural settings. Relatedly, cultural variation in hierarchy, relational obligations, and family roles may shape expectations regarding caregiving and parental involvement (28–30). For example, collectivist cultural orientations and greater acceptance of hierarchical relationships may influence expectations regarding parental involvement, caregiving, and relational responsibilities, thereby affecting how supportive childhood experiences are perceived and recalled (25). Furthermore, cultural competence frameworks emphasize that the significance of a given experience cannot be assumed to be equivalent across cultural settings because meanings are constructed through ongoing interactions between individuals and their sociocultural environments (26). Consequently, measures developed in Western populations may not fully capture culturally specific manifestations of support, care, and relational security in non-Western contexts. The need for cross-cultural examination is further underscored by evidence that the expression and meaning of childhood warmth and care vary across cultures. In Western settings, supportive experiences are often conveyed through explicit verbal affirmation and affective expression (21, 22), whereas in East Asian contexts, including South Korea, support may be expressed more indirectly through instrumental provision, educational investment, and relational consistency (23, 24). For example, Chao (31) states that parental “training” in many East Asian families reflects a culturally embedded form of care and involvement that may not be adequately captured by Western conceptualizations of parental warmth. Similarly, relational security may be fostered through fulfillment of family obligations, guidance, and long-term commitment rather than through overt emotional affirmation alone (28, 29, 31). Such culturally patterned expressions of care may influence how positive relational experiences are perceived, internalized, recalled, and integrated into stress-regulatory and self-evaluative processes, highlighting the importance of considering cultural context when examining associations between PCEs and health-related behaviors. Moreover, relational resources are socially patterned by socioeconomic position and family structure. The sociocultural context surrounding eating-related behaviors may also differ meaningfully across Asian populations. Research on eating disorders in Asia suggests that risk is influenced not only by thin-body ideals but also by rapid social change, Westernization, media exposure, globalization, migration, and country-specific social pressures (32). In South Korea specifically, appearance-oriented social norms, high levels of body surveillance, and strong social and academic performance expectations may contribute to body dissatisfaction and eating-related concerns (33). In societies characterized by pronounced appearance norms (34), intense academic competition and distinct parent-child relational hierarchies (24, 35), early relational experiences may intersect with sociocultural pressures in shaping vulnerability to eating-related behaviors. These contextual factors suggest that the developmental significance and functional meaning of PCEs may vary across sociocultural contexts and that associations observed in Western population may not necessarily generalize to other settings. Together, these considerations suggest that PCEs may vary not only in prevalence but also in developmental salience and functional meaning across sociocultural contexts. To address this gap, the present study examines associations between cumulative PCEs and multiple domains of DEABs among Korean college students. Drawing on life-course perspectives that emphasize the potential long-term influence of early psychosocial environments on later health and behavior, we hypothesized that lower levels of cumulative PCEs would be associated with a greater prevalence of DEABs. Consistent with prior studies reporting dose-dependent associations between PCEs and health-related outcomes, we further explored whether associations varied across levels of cumulative PCE exposure.

## Methods

### Participants and recruitment

The data used in this study were derived from a pilot survey designed to examine the distribution of positive childhood experiences and their potential associations with eating-related behaviors among Korean college students. Participants for this study were undergraduate students aged 18 years or older who were currently enrolled at a large public university in South Korea. Data were collected between April 2025 and June 2025. Participants were recruited through online advertisements and offline bulletin board postings distributed throughout the university. Recruitment was not conducted through specific courses or classroom. Eligibility was limited to currently enrolled non-pregnant undergraduate students aged 18 years or older. No additional exclusion criteria were applied, consistent with population-based research examining DEABs among college students. Participants received a modest incentive (approximately USD $10 equivalent) upon completion. The study protocol was reviewed and approved by an institutional review board (IRB No. 34, March 2025), and electronic informed consent was obtained prior to participation. All procedures were conducted in accordance with the declaration of Helsinki. A total of 309 students initiated the survey. Of these, 287 students completed the survey. After excluding participants with missing data on key exposure, outcome, or covariate variables (12 missing DEABs items; two missing covariates), the final analytic sample consisted of 273 students with complete data. Participants with missing data were excluded using complete-case analysis. Overall missingness was low (< 5%) and did not differ meaningfully across PCE tertiles (data not shown), suggesting minimal risk of differential bias due to missing data. Because recruitment occurred through public advertisements and bulletin board postings, the number of students exposed to recruitment materials was unknown and a response rate could not be calculated.

### Measures

#### Positive childhood experiences (PCEs)

Positive childhood experiences (PCEs) were assessed using the 10-item Benevolent Childhood Experiences (BCE) Scale (15), which retrospectively assesses positive experiences occurring before age 18 years. Because the BCE scale was designed to assess the accumulation of diverse protective experiences rather than a single latent construct, summed scores were treated as a cumulative index of positive childhood experiences, consistent with prior PCE research (15). Therefore, throughout this manuscript, scores derived from the BCE Scale are interpreted as indicators of cumulative positive childhood experiences (PCEs). For clarity, the BCE Scale was used solely as the measurement instrument, whereas the term PCEs is used throughout the manuscript to refer to the broader construct being assessed. The BCE scale captures relational, emotional, and environmental assets, including experiences such as having supportive caregivers, positive peer relationships, opportunities for enjoyment, and feelings of safety and belonging (15). The 10 PCE items are provided in Supplementary Table S1. Consistent with the original BCE framework, these items were dichotomized and summed to generate a cumulative PCE score (range: 0–10) following the original scoring protocol, with higher scores indicating greater exposure to positive childhood experiences, rather than weighted according to individual experience type (15). Consistent with the conceptual framework of the BCE scale, positive childhood experiences are distinct form, and not merely the absence of adverse childhood experiences (ACEs). Prior research suggests that positive and adverse childhood experiences represent related but distinct dimensions of childhood environments and may independently influence health and wellbeing across the life course (15, 16, 36, 37). Although the BCE scale was originally developed and validated primarily in Western populations, a recent study translated and culturally adapted the BCE scale for Korean college students using a rigorous translation and back-translation procedure (38). In that study, multiple bilingual researchers independently translated the original English items into Korean, followed by back-translation and evaluation of semantic equivalence between the original and translated versions. The Korean version demonstrated satisfactory psychometric properties, including support for the original one-factor structure and evidence of convergent and criterion validity (38). In addition, studies conducted in Chinese community samples have reported acceptable reliability and predictive validity, further supporting the applicability of the scale in East Asian populations (20, 39). Internal consistency in the present sample was modest (Cronbach alpha = 0.66), consistent with prior research and reflective of the multidimensional nature of relational experiences captured by the scale. To examine potential non-linear associations, PCE scores were categorized into tertiles (T1 = lowest; T3 = highest exposure), allowing examination of potential accumulation effects. Because categorization may result in some loss of information, sensitivity analyses were conducted using the total PCE score as a continuous variable.

#### Disordered eating attitudes and behaviors (DEABs)

DEABs were assessed using the items from the Questionnaires on Eating and Weight Patterns-5 (QEWP-5), a self-report instrument developed to assess eating-related attitudes and behaviors associated with eating disorder psychopathology (40). Consistent with prior epidemiologic research, five DEABs, including overeating, binge eating, anxiety-associated symptoms of binge eating, unhealthy weight control behaviors, and body weight and shape concerns were examined separately (14, 41). Overeating was defined as reporting episodes during the past 3 months in which participants ate an unusually large amount of food. Binge eating was defined as overeating accompanied by a perceived loss of control over eating. Anxiety-associated symptoms of binge eating were defined as endorsing emotional distress (e.g., guilt, embarrassment, or anxiety) related to binge eating episodes. Unhealthy weight control behaviors were assessed by self-reported engagement in at least one unhealthy weight control behaviors during the past 3 months, including behaviors such as fasting, self-induced vomiting, misuse of laxatives or diuretics, or other compensatory behaviors. Body weight and shape concerns were assessed using QEWP-5 items evaluating the extent to which body weight or shape influenced self-evaluation. Each outcome was dichotomized (0 = absent, 1 = present) using established QEWP-5 criteria and procedures consistent with prior epidemiologic studies using similar operational definitions (14, 41). Because these indicators represent conceptually distinct behavioral and attitudinal constructs rather than manifestations of a single latent construct, outcomes were analyzed separately rather than combined into a composite score. Internal consistency across DEABs was low (Cronbach alpha = 0.51). However, low internal consistency was expected because the DEAB indicators were not intended to measure a single underlying construct. Accordingly, Cronbach's alpha is of limited interpretive value in this context and was reported only for descriptive purposes.

Although the QEWP-5 has demonstrated utility in identifying eating disorder-related behaviors and symptoms in epidemiologic and clinical research, the specific QEWP-5 items used in this study have not been formally validated among Korean college students. Therefore, findings should be interpreted with caution, and additional psychometric evaluation of the instrument in Korean young adult populations is warranted.

### Covariates

Covariates were selected *a priori* based on theoretical relevance and prior literature. These included age (years), sex (male/female), academic classification (year in school), maximum parental educational attainment (less than high school, some college, bachelor's degree or higher), and body mass index (BMI), all self-reported. Age, sex, and academic classification were included to account for potential developmental differences within the college population. Parental education was included as a proxy for childhood socioeconomic position, reflecting structural and resource-based dimensions of early life context that may shape both exposure to relational assets and eating-related outcomes. BMI was included because body weight status has been consistently associated with DEABs and may also be related to childhood social and familial experiences. Although prior research suggests that ACEs and PCEs are related but distinct constructs, and childhood adversity may influence DEABs (10, 12, 13, 42), given that ACEs were not assessed in the present study, ACEs was not included in the adjusted models.

### Data analysis

Modified Poisson regression with robust standard errors was used to examine associations between PCEs and DEABs and to estimate prevalence ratios (PR) and 95% confidence intervals (CIs). This approach was selected to directly estimate PRs given the non-rare prevalence of DEAB outcomes and to avoid overestimation associated with odds ratios in cross-sectional data and logistic regression models (43, 44). Robust standard errors were used in all models.

PCEs were categorized into tertiles and modeled with the highest tertile (T3) as the referent group. Tertiles were used to facilitate interpretation and to examine potential non-linear associations across levels of PCEs. Because categorization of continuous variables may result in loss of information and reduced statistical power (45, 46), sensitivity analyses were additionally conducted using the continuous PCE score. All models were adjusted for age, sex, academic classification, parental education, and BMI. Linear trend across tertiles was assessed by modeling the ordinal tertile PCE variable as a continuous term. Outcome frequencies and prevalence estimates were examined across PCE tertiles to evaluate event distributions and potential sparse-data concerns. All models converged without warnings or estimation difficulties, no sparse outcome cells were identified that would be expected to compromise model stability. Because this study was exploratory and evaluated multiple related DEAB outcomes, no formal adjustment for multiple comparisons was applied. Rather than focusing on statistical significance testing, interpretation emphasized the magnitude, direction, and precision of effect estimates as reflected by prevalence ratios (PRs) and 95% CIs (47). Statistical significance was defined as *p* < 0.05; however, interpretation emphasized effect estimates and confidence intervals rather than statistical significance alone. All analyses were conducted using SAS version 9.4 (SAS Institute Inc., Cary, NC, USA).

## Results

### Baseline characteristics of the analytical sample

The mean age of participants was 22.7 ± 2.2 years and 71.4% (*n* = 195) were female college students. Approximately 84.2% reported that their parents' maximum educational attainment was a bachelor's degree or higher. The mean BMI was 21.9 ± 3.3 kg/m^2^. Participant characteristics by PCE tertiles are presented in Table 1.

**Table 1:** Baseline sociodemographic characteristics of analytical sample (*n* = 273).

Sociodemographic characteristics	T3 (PCE score range: 9–10; *n* = 114)	T2 (PCE score: 8; *n* = 64)	T1 (PCE score range: 0–7; *n* = 95)	*p*-value
Age (M_age_ ± SD)	22.7 ± 2.1	22.4 ± 2.0	22.9 ± 2.5	0.49
Sex				0.51
Female	79 (69.3)	44 (68.8)	72 (75.8)	
Male	35 (30.7)	20 (31.3)	23 (24.2)	
Parental maximum educational attainment				0.11
≤ High school	15 (13.2)	8 (12.5)	20 (21.1)	
College	69 (60.5)	43 (67.2)	56 (59.0)	
Grad school	30 (26.3)	13 (20.3)	19 (20.0)	
BMI (kg/m^2^)	22.2 ± 3.4	22.8 ± 3.1	21.4 ± 3.1	0.25

### Associations between PCE tertiles and DEABs

Prevalence ratios for overeating, binge eating, and body weight and shape concerns were generally above 1.0 among students in the lower PCE tertiles, although confidence intervals were wide and included the null value. Compared with students in the highest PCE tertile (T3), those in the lowest tertile (T1) showed a higher estimated prevalence of overeating (PR =1.22, 95% CI = 0.82–1.81). Similar patterns were observed for binge eating and body weight and shape concerns. Those in T2 and T1 each had 1.32 times (95% CI = 0.69–2.54) and 1.20 times (95% CI = 0.66–2.16) the prevalence of binge eating; College students in T2 and T1 had 1.55 times (95% CI = 0.89–2.71) and 1.53 times (95% CI = 0.92–2.56) the prevalence of body weight and shape concerns compared to those in T3, respectively. PCEs were not meaningfully associated with other DEABs assessed in this study (Table 2). Furthermore, tests for linear trend across tertiles were not statistically significant (all *p* for trend >0.10; Table 2). Thus while the point estimates were directionally consistent with a higher prevalence of these outcomes among students with fewer PCEs, the findings were imprecise and did not provide strong evidence of a graded association. Prevalence estimates for each DEAB outcome by PCE tertile are presented in Supplementary Table S2.

**Table 2:** Prevalence ratio (PR) and 95% confidence intervals of disordered eating attitudes and behaviors (DEABs) by PCE tertiles (*N* = 273).

Tertiles	Disordered eating attitudes and behaviors (PR, 95% CI)				
	Overeating	Binge eating	Anxiety-associated symptoms of binge eating	Unhealthy weight control behaviors	Body weight and shape concerns
T3 (ref) (*n* = 114)	1.00 (ref)	1.00 (ref)	1.00 (ref)	1.00 (ref)	1.00 (ref)
T2 (*n* = 64)	1.07	1.32	1.07	1.02	1.55
	(0.68–1.67)	(0.69–2.54)	(0.75–1.54)	(0.70–1.49)	(0.89–2.71)
T1 (*n* = 95)	1.22	1.20	1.13	1.04	1.53
	(0.82–1.81)	(0.66–2.16)	(0.82–1.56)	(0.74–1.45)	(0.92–2.56)
*p* for trend	0.32	0.55	0.44	0.83	0.10

Models adjusted for age, sex, academic classification, maximum parental educational attainment, and BMI.

### Sensitivity analyses for associations between continuous PCE scores and DEABs

Sensitivity analyses modeling PCEs as a continuous variable yielded results consistent with the primary analyses (Table 3). PCE scores were not significantly associated with overeating (adjusted PR = 0.94, 95% CI = 0.86–1.03), binge eating (adjusted PR = 1.00, 95% CI = 0.87–1.14), anxiety-associated symptoms of binge eating (adjusted PR = 0.97, 95% CI = 0.90–1.05), unhealthy weight control behaviors (adjusted PR = 0.99, 95% CI = 0.91–1.07), or body weight and shape concerns (adjusted PR = 0.90, 95% CI = 0.80–1.00). Sensitivity analyses modeling PCEs continuously yielded estimates that were similarly close to the null value and generally consistent with the primary analyses, suggesting that the overall pattern of findings was not materially influenced by categorization of PCE scores into tertiles.

**Table 3:** Sensitivity analyses of prevalence ratio (PR) and 95% confidence intervals of disordered eating attitudes and behaviors (DEABs) by PCE score (*N* = 273).

Disordered eating attitudes and behaviors (PR, 95% CI)				
Overeating	Binge eating	Anxiety-associated symptoms of binge eating	Unhealthy weight control behaviors	Body weight and shape concerns
0.94 (0.86–1.03)	1.00 (0.87–1.14)	0.97 (0.90–1.05)	0.99 (0.91–1.07)	0.90 (0.80–1.00)

Models adjusted for age, sex, academic classification, maximum parental educational attainment, and BMI.

## Discussion

The present study examined associations between cumulative PCEs and DEABs among Korean college students. Students in the lower PCE tertiles (T1 and T2) had elevated point estimates for binge eating and body weight and shape concerns relative to those in the highest tertile (T3). However, associations were imprecisely estimated and did not reach statistical significance. Furthermore, sensitivity analyses modeling PCEs continuously yielded largely null findings, suggesting limited evidence for a linear association between PCEs and DEABs in this sample of Korean college students. Therefore, the findings suggest these findings should be interpreted as exploratory and suggestive rather than as evidence of a clear association. Although the evidence was imprecise, the pattern of elevated point estimates was observed primarily for binge eating and body weight and shape concerns rather than uniformly across all DEAB domains. This pattern underscores the heterogeneity of eating-related behaviors and the multidimensional nature of DEABs. Certain behaviors, such as unhealthy weight control behaviors, may be shaped more by proximal sociocultural and peer influences than by early relational experiences. If confirmed in larger studies, these findings would be consistent with evidence that PCEs foster adaptive coping and lower psychological distress (16, 17), which could potentially reduce vulnerability to certain disordered eating attitudes and behaviors. These observed directions of the estimates may also be interpreted within resilience and stress-buffering frameworks (48, 49), which posit that early experiences characterized by warmth, safety, and predictability foster emotional regulation and stable self-worth (50). However, the present study was not designed to directly test these mechanisms.

From a life-course perspective, these findings suggest that early relational environments may shape vulnerability to specific eating-related behaviors in young adulthood (10, 13, 14). The absence of statistically significant dose-response trends limits conclusions regarding the functional form of any potential relationship between PCEs and DEABs. Although some associations appeared stronger among participants reporting lower levels of cumulative PCEs, these findings should not be interpreted as evidence of threshold effects. Because the present study relied on retrospective assessment of childhood experiences within a cross-sectional design, it cannot distinguish threshold effects from alternative explanations, including accumulation processes, measurement limitations, recall bias, or residual confounding. Rather, the observed patterns provide preliminary evidence that is broadly consistent with life-course hypothesis of childhood experiences related to later eating related behaviors. Importantly, a novelty of this study is that it examined the associations between PCEs and DEABs within a non-Western sociocultural context. Although the BCE Scale captures broadly defined relational assets such as safety, support, and caring adults (15), the enactment and interpretation of support may differ in Korean families compared to Western contexts in which the scale was developed. Indirect expressions of care, academic investment, and relational duty, which may be more common in non-Western cultures (23, 24) may not be fully captured by existing items, potentially influencing observed magnitudes. These findings underscore the importance of continued cultural validation and measurement refinement. Childhood environments in Korea are often characterized by less overt parental warmth and more indirect expressions of support (51), which may shape how PCEs are perceived and internalized. Moreover, DEABs may not represent a unified construct in this population, as reflected by low internal consistency across indicators and heterogeneity influenced by appearance-oriented norms (52), academic pressures (24), and sociocultural expectations regarding body image (34). These contextual factors may attenuate cumulative associations when DEABs are examined collectively. Consistent with this interpretation, PCEs were not associated with anxiety-associated symptoms of binge eating or unhealthy weight control behaviors, which may be more strongly influenced by proximal stressors (53) or sociocultural normalization of restrictive practices (24, 34). Taken together, these exploratory findings suggest that any relationship between PCEs and eating-related outcomes may be context-specific and domain dependent rather than uniformly observed across eating-related outcomes. However, given the imprecision of the estimates, these interpretations should be considered tentative pending replication in larger and more diverse samples.

Findings from this study should be interpreted in light of several limitations. The cross-sectional design precludes causal inference, and temporality cannot be established. The modest sample size may have limited power to detect small associations. Consequently, several estimates were imprecise, as reflected by wide confidence intervals that included the null value, and the study may have been underpowered to detect dose-response trends across PCE tertiles. Measurement reliability was modest, though consistent with the multidimensional nature of both PCEs and DEAB constructs. Additionally, although the QEWP-5 has been widely used to assess eating disorder-related attitudes and behaviors in epidemiologic and clinical research, formal validation of the Korean version among Korean college students remains limited. Consequently, the extent to which the instrument captures constructs such as body weight and shape concerns, anxiety-associated binge eating symptoms, and unhealthy weight control behaviors with equivalent validity in this cultural context is not fully established. Future studies should evaluate the psychometric properties and cultural applicability of the QEWP-5 among Korean young adult populations. Generalizability is limited by the convenience sample from a single university and an unbalanced sex distribution, and self-reported data may have introduced recall or reporting bias. Additionally, although parental education was included as a proxy for childhood socioeconomic position, future work should more directly examine how relational assets and structural disadvantage intersect. Life-course processes are socially patterned, and access to supportive relational environments may itself reflect broader socioeconomic stratification. Larger studies with greater socioeconomic heterogeneity are needed to evaluate potential effect modification by childhood socioeconomic position. Another limitation of this study is that adverse childhood experiences (ACEs) were not assessed. Although ACEs and PCEs represent distinct dimensions of childhood environments, they are often correlated and may independently or jointly influence later health and behavioral outcomes (10, 12, 41). Considering extensive evidence indicates that childhood adversity is associated with adverse mental and physical health outcomes across the life course (42, 54), residual confounding by unmeasured childhood experiences may have influenced the observed associations between PCEs and DEABs. Future studies should assess both positive and adverse childhood experiences to better disentangle their independent and potentially interactive associations with eating-related outcomes.

Despite these limitations, this study provides preliminary evidence regarding positive childhood experiences and eating-related outcomes in a non-Western young adult population. Although the study did not provide strong evidence of graded associations, elevated point estimates for binge eating and body weight and shape concerns among college students with lower PCEs suggest these a domains may warrant further investigation. Future research should employ longitudinal studies and culturally sensitive measures to determine whether the suggestive patterns observed in this study reflect true associations and to clarify how positive childhood experiences may influence eating- related trajectories across sociocultural contexts.

The present findings have implications for research and clinical practice. For researchers, the results underscore the importance of incorporating both adverse and positive childhood experiences into etiologic models of disordered eating, particularly in non-Western contexts. Although replication is needed, clinicians working with college students may consider assessing early relational strengths, in addition to adversity, when seeking a more comprehensive understanding of factors that could be related to eating-related concerns.

## Data Availability

The raw data supporting the conclusions of this article will be made available by the authors, without undue reservation.
