# Supplementary material for: Associations of positive childhood experiences with disordered eating attitudes and behaviors among Korean college students
Source: Front Public Health. 2026 Jul 15;14:1817868. doi: 10.3389/fpubh.2026.1817868 (PMC13415693; doi:10.3389/fpubh.2026.1817868)
Supplement: Supplementary file 1 [file Table_1.docx]

**Supplemental Table 1. Verbatim Items of Positive Childhood Experiences assessed from the Benevolent Childhood Experiences Scale**

| Item | English | Korean |
| --- | --- | --- |
| 1 | Did you have at least one caregiver with whom you felt safe? | 당신에게 안정감을 준 양육자가 적어도 한 명 있었습니까? |
| 2 | Did you have at least one good friend? | 친한 친구가 한 명이라도 있었습니까? |
| 3 | Did you have beliefs that gave you comfort? | 위안이 되는 믿음 (좌우명, 종교, 신념 등)이 있었습니까? |
| 4 | Did you like school? | 당신은 학교 생활을 좋아했나요? |
| 5 | Did you have at least one teacher who cared about you? | 당신에게 애정 어린 관심을 주는 선생님이 한 명이라도 있었습니까? |
| 6 | Did you have good neighbors? | 주변에 좋은 이웃들이 있었나요? |
| 7 | Was there an adult (not a parent or the person from item 1) who could provide you with support or advice? | (앞서 응답한 양육자/보호자를 제외하고) 당신에게 지지와 조언을 줄 수 있는 어른이 있었습니까? |
| 8 | Did you have opportunities to have a good time? | 좋은 시간이었다고 생각되는 추억이 있었습니까? |
| 9 | Did you like yourself or feel comfortable with yourself? | 당신 스스로를 좋아하거나 자신에게 편안함을 느꼈습니까? |
| 10 | Did you have a predictable home routine, like regular meals and a regular bedtime? | 가정에서는 예측 가능한 루틴 (규칙적인 식사 시간, 취침 시간 등)이 있었습니까? |

**Supplemental Table 2. Crude prevalence of disordered eating attitudes and behaviors (DEABs) by positive childhood experiences (PCE) tertiles (N=273)**

| **Outcome** | **T3 (n=114)** | **T2 (n=64)** | **T1 (n=95)** |
| --- | --- | --- | --- |
| Overeating | 52 (45.6%) | 30 (46.9%) | 50 (52.6%) |
| Binge eating | 21 (40.4%) | 16 (53.3%) | 26 (52.0%) |
| Anxiety-associated symptoms of binge eating | 79 (69.3%) | 47 (73.4%) | 74 (77.9%) |
| Unhealthy weight control behaviors | 76 (66.7%) | 43 (67.2%) | 65 (68.4%) |
| Body weight and shape concerns | 27 (23.7%) | 23 (35.9%) | 34 (35.8%) |
